# Gender Specific Differences in the Pros and Cons of Smoking among Current Smokers in Eastern Kentucky: Implications for Future Smoking Cessation Interventions

**DOI:** 10.3390/ijerph5040230

**Published:** 2008-12-08

**Authors:** Dana A. Hazen, David M. Mannino, Richard Clayton

**Affiliations:** 1 University of Kentucky College of Public Health, 121 Washington Avenue Lexington, KY 40536, USA; E-mail: danaalainehazen@gmail.com (D. A. H.); 2 Department of Preventive Medicine and Environmental Health University of Kentucky College of Public Health and Division of Pulmonary, Critical Care and Sleep Medicine University of Kentucky College of Medicine, 121 Washington Avenue Lexington, KY 40536, USA; E-mail: clayton@email.uky.edu (R. C.)

**Keywords:** Transtheoretical model, Decisional-balance, Stage of change, Smoking, Gender

## Abstract

This study investigated gender differences in the perceived “pros” and “cons” of smoking using the constructs of decisional balance (DB) and stage of change from the Transtheoretical Model. The population distribution for stage of change among a population-based, cross-sectional survey of 155 current smokers over 40 years was: precontemplation (22.6%), contemplation (41.9%), preparation (35.5%). Results of stepwise regression models indicated significant gender differences in DB were in the preparation stage of change; scores on the DB measure increased 3.94 points (95% CI: 1.94, 5.93) for male smokers. Interventions targeting the “pros” and “cons” of smoking may need to be gender specific.

## 1. Introduction

Cigarette smoking is the major risk factor for the development of chronic obstructive pulmonary disease (COPD), the 5^th^ leading cause of morbidity and mortality in the United States [1]. Data from the Third National Health and Nutrition Examination Survey (NHANES III) estimate that approximately 24 million U.S. adults have evidence of impaired lung function and the number of COPD related hospitalizations and deaths continue to rise. The COPD mortality rate nearly tripled from 1980 – 2000 among women as a result of increased smoking rates among women that increased significantly decades earlier [2, 3].

The Commonwealth of Kentucky has the highest rates of smoking in the country; nearly one-third of adults currently smoke. Furthermore, the percentage of females who smoke in Kentucky has increased steadily in the past three years [4]. Results from the Burden of Lung Disease (BOLD) global initiative estimate the prevalence of COPD among long-time, heavy smokers to be 22% in men and over 29% in women in the eastern region of the state [5]. Successful smoking cessation can stop the accelerated decline of lung function associated with COPD within 5 years [6, 7]. Furthermore, lung function improves within the first year of cessation [7], and evidence suggests that women gain a greater percentage of lung function back than men [8]. The Transtheoretical Model (TTM) [9, 10] is widely used in the literature to measure intent to stop smoking. The model includes four primary constructs that are inter-related: stages of change, processes of change, self-efficacy and decisional balance [9,10,11]. Movement through the stages of precontemplation (not thinking of quitting), contemplation (thinking of quitting within 6 months), preparation (thinking of quitting within 30 days), action (successful quit attempt in the past 6 months) and maintenance (sustained quitting over 6 months) is cyclical, as smokers may regress to earlier stages. Self-efficacy is defined as the temptation to perform a behavior and the situation-specific confidence to refrain from the behavior [12, 13]. Processes of change involve both the thought and emotional (experiential) and action-oriented activities (behavioral) people perform when getting ready to quit smoking [14].

The construct of Decisional Balance (DB) relates to the cognitive activity of weighing the positive and negative consequences, or “pros” and “cons”, of a specific behavior and is the focus of this study [15]. DB varies as a function of stage of change. The “pros” of smoking decrease while the “cons” increase as smokers move through the stages [16–20] with the cross-over between the “pros” and “cons” occurring in the contemplation or preparation stage [18]. Recent evidence suggests that the perceived “cons” of smoking play an important role during the earlier stages of change [21–23] while the changes in the perceived “pros” are associated with movement into the action and maintenance stages [12, 16, 24, 25].

Few studies find significant gender differences in intention quit [26]; however, female smokers report significantly more “pros” and “cons” to smoking than their male counterparts [26, 27]. Women are more likely to perceive weight control and the reduction of negative affect as a “pro” to smoking behavior [28–30]. Women are also less concerned about the “cons” of smoking related to health problems and are more motivated to quit by means of immediate reinforcement [31]. One study by Macnee & McCabe found that only the perceived “cons” of smoking differ between men and women who are in the early stages of change [23]. There are currently no studies evaluating differences in decisional balance between male and female smokers in each individual stage of change.

Therefore, the purpose of this study was to investigate gender differences in the perceived “pros” and “cons” of smoking in a high-risk population of current smokers. More specifically, the author hypothesized that gender differences in overall decisional balance are found across all stages of active smoking after controlling for smoking history variables previously demonstrated to affect decisional balance [32].

## 2. Experimental Section

### 2.1. Study Participants

This study is a secondary analysis of data from the Burden of Lung Disease (BOLD) study in Eastern Kentucky; the design and rationale for this study are reported elsewhere [33, 34]. Recruitment of participants involved using a random-digit dial telephone survey, and all agreeable telephone respondents were given the minimal data questionnaire [5]. Individuals who were 40 years of age or older qualified for the study. The overall participation rate was 14%. Of the 575 individuals who agreed to participate, 346 (60%) reported smoking during their lifetime. Only current smokers were able to answer the items on the DB Questionnaire due to the format of each question, limiting the analysis to the 158 current smokers. Three of the participants were missing data, leaving 155 individuals in the final analyses (so the prevalence of current smoking is 27%).

### 2.2. Data Collection

All eligible participants scheduled a visit to one of the study sites (local satellite offices of the University of Kentucky) and when necessary, participants had the survey administered in their homes. Survey questions were read aloud to participants and informed consent was obtained from each participant prior to participation in the study [5]. Participants received $30.00 Walmart gift cards for their time participating in the study, each questionnaire took approximately one hour to complete. The Institutional Review Board at the University of Kentucky approved this study.

### 2.3. Measures

#### 2.3.1 Demographic Variables and Smoking History

A single questionnaire assessed demographic information such as age, gender, years of education, and occupational status. Current smoking levels, age of smoking initiation, number of previous quit attempts, exposure to passive smoke and a series of questions evaluating prior physician advice to quit smoking were also included on the questionnaire. The calculation for the pack-years variable was the average number of cigarettes smoked per day divided by 20 and multiplied by the number of years the participant smoked. The pack-years smoked variable accounts for both duration of smoking as well as frequency and may be an indicator of nicotine addiction levels.

#### 2.3.2 Stages of Change

Stage of Change was measured using the Transtheoretical Model developed by Prochaska and DiClemente [9]. All smokers in this study were current smokers, thus excluding the action and maintenance stages from further analysis. One of the questionnaires contained the question “Are you seriously thinking of quitting smoking?”, and participants were categorized into one of the stages according to their response: “No, not thinking of quitting” (precontemplation); “Yes, within the next 6 months” (contemplation); or “Yes, within the next 30 days” (preparation).

#### 2.3.3 Decisional Balance

A short form of the Decisional Balance (DB) Inventory developed by Velicer, *et al.*[15] was composed of a 3-item scale weighing the perceived social “cons” (e.g. “People think I’m foolish for ignoring the warnings about cigarette smoking”) and 3-items weighing the coping “pros” (e.g.“Smoking helps me concentrate and do better work”) to smoking for a total of 6-items. A 5-point Likert scale ranging from “not important” to “extremely important” rated each item. Scores for the cons subscale subtracted from scores for the pros subscale produced an aggregate DB score. Higher scores on the DB scale indicate a positive balance to smoking, or more importance to the “pros” than “cons” of smoking. Cronbach’s alpha, measuring the internal reliability between items on each subscale, was 0.70 for the “pros” of smoking and 0.58 for the smoking “cons” subscale (overall Cronbach’s 0.54) (because alpha is the average of all the split halves the limited number of items in these scales makes the reliability estimates from alpha possibly unstable). The use of this instrument has been validated in other studies [22, 23], and multiple studies have demonstrated content validity between the commonly used Smoking Consequences Questionnaire (SCQ-A) and longer versions of the DB scale [32, 35].

### 2.4. Data Analysis

A series of t-tests, chi-square analyses examined bivariate relationships between socio-demographic, smoking status variables, stage of change, and decisional balance subscale scores. Pearson’s correlations assessed relationships between socio-demographic and smoking history variables. Step-wise linear regression models investigated multivariate relationships between aggregate DB scores, gender and other smoking characteristics. The statistical analyses used an acceptance level of *p* <0.05. SAS 9.1 software (SAS Institute; Cary, NC) was used to perform all the statistical analyses.

## 3. Results and Discussion

### 3.1. Demographics

This sample of active, current smokers consisted of 90 females (58%) and 65 males (42%). Ninety-eight percent of the sample was Caucasian, with 2 individuals characterizing themselves as “other”. The sample ranged in age from 40 – 81 years, with a mean age of 53.4 years (SD 8.9 years). Over 67% of the sample had at least a high school education. More than 1/3 (37%) of the sample reported working for income during the past year, with 12% of the respondents unable to work due to health problems. Seventeen percent of the sample reported a previous diagnosis of emphysema, and 12% of the sample reported a diagnosis of chronic bronchitis. Interestingly, only 12% of the sample reported ever being diagnosed with COPD.

### 3.2. Smoking History

The sample smoked an average of 25 cigarettes per day or a little more than a pack per day, with one participant reportedly smoking 80 cigarettes per day. The mean smoking duration was 46.2 pack-years. Men were heavier smokers, smoking significantly more cigarettes per day than women (*t* (155) = − 2.527; *p* < 0.012) for a greater number of years (*t* (155) = −2.626; *p* < 0.0095). When looking at prior quit attempts, the median number of quit attempts in the previous year was only 1, ranging from 0 to 60. In addition, 87% of the sample reported receiving physician advice to quit smoking, with 66% receiving such advice within the past 12 months. Over half of the sample reported current passive smoke exposure in the home during the previous two weeks.

### 3.3. Stage of Change

To characterize the sample according to stage of change, 22.6% of the participants were in the precontemplation stage, 41.9% were in the contemplation stage of change and 35.5% of smokers were in the preparation stage of change. There were no significant relationships between stage of change and age, years of education or work status. Chi-square analyses revealed no significant differences in gender for the early stages of change (χ^2^ = 4.502; *p* <0.10); however, there were differences for the smoking history variables. The findings from a series of independent t-tests showed precontemplators made fewer quit attempts in the previous year than contemplators (*t* (100) = 2.941; *p* <0.005) and preparers (*t* (90) = 2.936; *p <0.0*18). Precontemplators also smoked a greater number of pack-years than contemplators (*t* (100) = −2.308; *p* <0.02). There were no differences in pack-years between precontemplators and preparers (t (90) = −1.35; p <0.17) or contemplators and preparers (t (120) = −1.3; p <0.19). Table 1 summarizes demographic and smoking history variables according to gender and stage of change.

### 3.4. Decisional Balance by Stage of change

**Figure 1:**
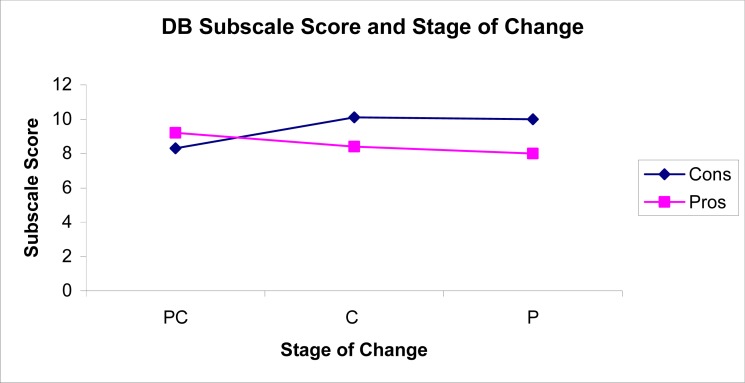
Mean Subscale Scores by Stage of Change.

A series of independent t- tests compared DB subscale scores across the stages of change. Scores for the Pros subscale and Cons subscale were not related (*r* = −0.065; *p* <0.639), and only the scores on the “cons” subscale differed across the stages of change. Precontemplators scored significantly lower on the DB cons subscale when compared to contemplators (*t* (100) = 3.18; *p* <0.002) and preparers (*t* (90) =2.797; *p* <0.0063) as they perceived fewer cons to smoking than contemplators and preparers. There was no difference in scores between smokers who intended to quit in 1 month (preparation stage) or in 6 months (contemplation stage) (*t* (120) = −0.138; *p* <0.8906). Overall decisional balance was positive among precontemplators (M = 0.91; SD = 3.66) and negative for contemplators (M = −1.7; SD = 3.78) and preparers (M = −2.0; SD = 4.7), indicating the shift in decisional balance may occur in the contemplation stage (Figure 1).

### 3.5. Gender differences in Decisional Balance

There were significant gender differences for aggregate scores on the decisional balance scale (*t* (155) = −3.517, *p* < 0.0006). Women (M = −2.18; SD = 4.27) had significantly lower scores on the scale than men (M = 0.15; SD = 3.83). Gender differences were in the scores of the “cons” DB subscale but not the “pros” subscale score (*t* (155) = 3.573, *p <* 0.0005), with women perceiving more “cons” to smoking than men (Table 2). When looking at responses to each item of the DB “cons” subscale, women reported being more embarrassed to smoke (*t* (155) = 3.806; *p* < 0.0024), and conscious or “foolish” for ignoring the dangers of cigarette smoking (*t* (155) = 2.791; *p* < 0.0059). Women did not differ from men in the opinion that their smoking bothered other people (*t* (155) = 1.697; *p* < 0.09). Although there were no differences between men and women in the “pros” subscale score, men did smoke more to aid in concentration than women (*t* (155) = −2.429; *p* <0.0163). There were no differences in perceptions that smoking induces relaxation (*t* (155) = − 0.752; *p* <0.4529) or relieves tension (*t* (155) = − 0.099; *p* < 0.9211) between men and women (Table 2).

### 3.6. Results of Regression Models

**Figure 2:**
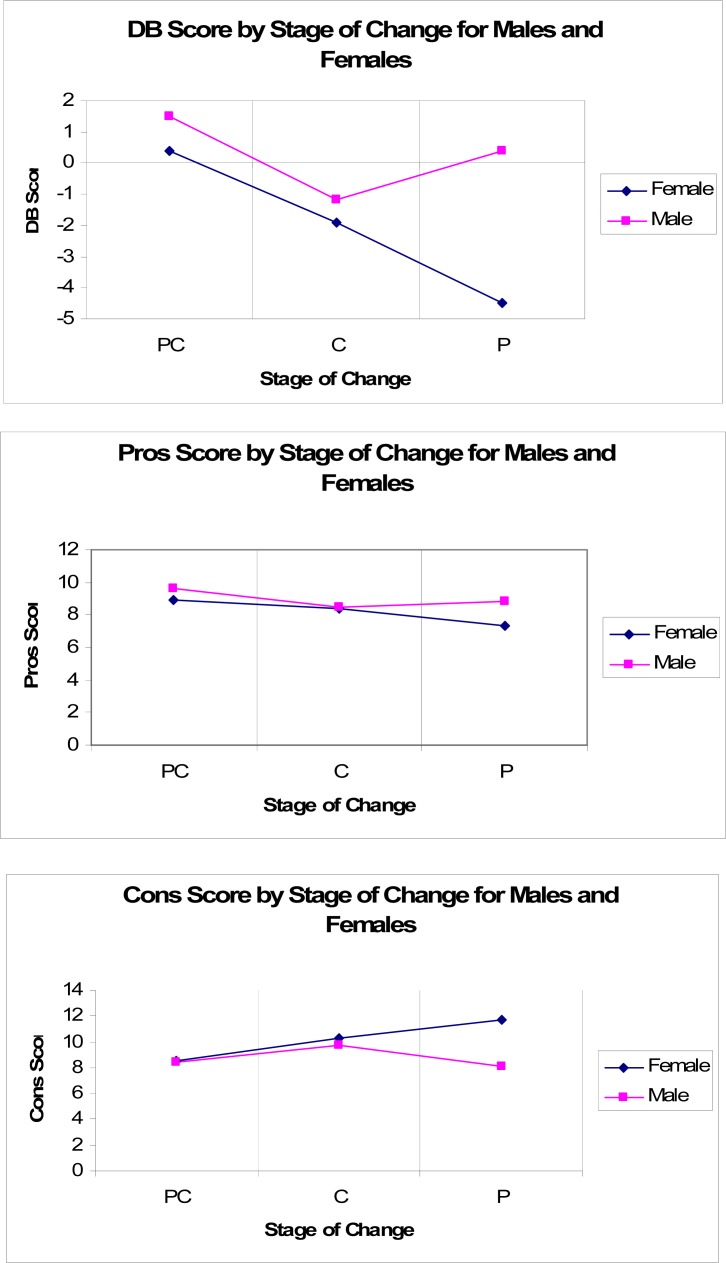
Decisional Balance across the Stages of Change for Males and Females.

A series of step-wise regression models evaluated the unique effect of gender on mean aggregate DB scores in each stage of change. A correlation matrix assessed multicollinearity between socio-demographic and smoking history variables before entering the variables into the step-wise regression model. There were no statistically significant relationships between any of the sociodemographic variables listed in Table 1 and DB scores. Aggregate scores on the DB scale were moderately correlated with the pack-years (*r* = 0.35; *p* <0.001) and weakly correlated with number of prior quit attempts (*r* = −0.19; *p* <0.001). Heavier smokers and those with fewer quit attempts had a more positive decisional balance. The relationship between scores on the DB “cons” subscale and gender was moderated by stage of change as there were significant gender differences in aggregate DB scores in only the preparation stage of change (*F* (3, 51) = 14.11; *p* <0.0001) after adjusting for smoking history variables. Neither the smoking history variables nor gender reached significance at the alpha level *p* < 0.05 in the regression models for the contemplation and precontemplation stages. Interestingly, men showed a positive decisional balance in both the precontemplation and preparation stages (Figure 2).

Variables were included in the model in the order of significance for the *t* statistic. Gender, number of prior quit attempts in the past year, and pack-years smoked explained 45% of the variance in aggregate DB scores (adjusted *R^2^=0.4215; F* (3, 51) = 14.11; *p* <. 0001). At Step 1, gender explained a significant 27.4% (adjusted *R^2^* = 0.2603) of the variance in DB scores, with almost 40% (adjusted *R^2^* =0.3762) of the variance in DB scores explained by pack-years history and gender at Step 2. The number of lifetime quit attempts added an additional 5% to the model’s explanatory value in the final step (Table 3). P-values for the estimated beta coefficients provide strong evidence that each of the independent variables is significantly related to aggregate DB scores at the alpha level 0.05. Furthermore, the parameter estimate for gender suggests that mean aggregate DB scores are 3.94 points (95% CI: 1.94, 5.93) greater for men versus women during the preparation stage for any given number of pack-years smoked or number of prior quit attempts.

Among this sample of 155 middle-aged smokers, the average number of pack-years smoked was 46.2 years, indicating that this is a sample of long-time heavy smokers. Furthermore, the men were heavier smokers than women. Twenty-two percent, 41% and 35% of the sample were in the precontemplation, contemplation and preparation stages of change respectively. The high percentage of smokers who intended to quit smoking within the next 6 months indicates this population of smokers differs from smokers in other areas of the Appalachian region. Results by Macnee & McCabe found only 30% and 14% of smokers in the contemplation and preparation stages [23]. There were differences in smoking history variables across the stages of change in the number of lifetime quit attempts between smokers intending to quit and precontemplators and the number of pack-years smoked between precontemplators and contemplators. This finding that is inconsistent with the results of studies showing that cigarette consumption, but not lifetime quit attempts differs by stage of change or smoking status [20, 32].

### 3.7. Decisional Balance and Stage of Change

As expected, decisional balance varied across the stages of change, with significant differences in the perceived “cons” of smoking but not the “pros”. These results replicated the findings from previous studies, as the “cons” differed significantly between precontemplators and contemplators or preparers and less between contemplators and preparers [16, 20, 21, 22, 23]. Only the results of a few studies have demonstrated that “cons” and the “pros” differed monotonically across the stages [17, 24]. Differences in these results may be attributed to the socio-demographic characteristics of each sample or variations in the measurement of intention to quit smoking.

There were significant gender differences in decisional balance scores within the early stages of change. Female smokers, on average, had a more negative decisional balance than male smokers and were more likely to feel embarrassed or foolish for smoking. Men held stronger perceptions that smoking aids in concentration then women. Differences in aggregate scores were the result of the “cons” subscale, but not the “pros” subscale scores of the decisional balance measure. These findings are in agreement with those of Macnee & McCabe [23] but contrast findings from other studies in which there were gender differences in both the “cons” and “pros” to smoking [26, 27]. Contradictions in the results among studies could be the result of the type of “pros” and “cons” (i.e. social or coping) used in measurement. Results by Plummer *et al*. (2001) demonstrate that the coping “pros” differ significantly across each stage of change, but the social “pros” differ significantly only between precontemplators and smokers in the abstinence stages [36].

### 3.8. Gender, Stage of Change, and Decisional Balance

Contrary to the hypothesis, gender differences in the perceived “pros” and “cons” of smoking were specific only to smokers who intended to quit smoking within the next 30 days. Gender accounted for almost 30% of the variance in DB scores during the preparation stage independent of pack-years smoking history and number of previous quit attempts. Gender did not explain the variance in DB scores among smokers in the precontemplation and contemplation stages. The men in this sample reported a positive decisional balance in the preparation stage, which may indicate this group of smokers is at risk for relapse [37].

## 4. Conclusions

Findings from this study indicate that among current smokers in Eastern Kentucky, there are gender differences in the perceived “pros” and “cons” of smoking. Furthermore, stage of change may moderate the relationship between gender and decisional balance. Interventions targeting a change in the balance of pros and cons of smoking need to be gender specific. These preliminary results indicate that men using smoking as a coping mechanism, thus interventions focusing around healthier ways to cope with stress may benefit male smokers. Whereas women who are preparing to stop smoking may benefit from cessation counseling centering on the social “cons” to smoking and ways to improve self-image.

## Figures and Tables

**Table 1:** Demographics of study participants by stage of change category.

Demographic	Total Sample (n = 155)	Precontemplators (n = 35)		Contemplators (n = 65)		Preparers (n = 55)	

		Men	Women	Men	Women	Men	Women

Sex (% Female)	58.0^c^	45.8	54.2	32.3	67.7	50.9	49.1
Mean Age^b^	53.4 (8.9)	51.6 (8.8)	58.5 (9.8)	50.5 (7.4)	52.2 (9.9)	54.2 (8.4)	54.7 (7.2)
Race (% White)	98.7	93.8	100	100	97.7	100	100

Education (%)							

Elementary	4.5	6.25	10.5	0	4.6	3.6	3.7
Middle	29.7	18.8	36.8	23.8	20.5	39.3	40.7
High School	32.9	37.5	31.5	52.4	34.1	25	22.2
College	32.3	37.5	21.1	23.8	38.6	32.1	33.2
Unknown	0.6				2.3		
Employed (%)	36.8	37.5	26.3	42.9	40.9	39.3	29.6

Smoking history							

Avg smoke/day^b^	25.5 (12.5)	30.3 (15.1)	26.7 (11.5)	27.9 (13.7)	23.1 (11.2)	28.0 (11.1)	21.7 (13.5)
Pack-Years^b,d,f^	46.2 (26)	58.8 (36.1)	51.0 (23.9)	48.4 (23.2)	38.5 (23.4)	52.4 (19.8)	40.1 (29.3)
Smoke Quit Attempt^c,d,e^	1 (0–60)	0 (0–4)	0 (0–2)	0 (0–8)	1 (1–50)	1.5 ( 0–60)	1 (1–60)
Physician Advice (%)	88	87.5	89.4	95.2	90.9	85.7	81.5
Passive Smoke (%)	52.9	68.8	47.3	57.1	52.3	46.4	51.9

*Note.* Due to missing data the sample size equaled 155. Smoke Quit Attempt, Lifetime Quit Attempts

a Percent female

b Mean (standard deviation)

c Median (range)

d Significant difference between PC and C based on results of t-test; p <0.05

e Significant difference between PC and P based on results of t-test; p <0.05

f Significant difference between men and women based on results of t-test; p <0.005

**Table 2:** Mean Decisional Balance, Pros and Cons Subscale Scores by Gender.

Decisional Balance ^a,b^	Total Sample (n = 155)	Female Smokers (n = 90)	Male Smokers (n = 65)
Aggregate ^c^	−1.20 (4.24)	−2.18 (4.27)	0.15 (3.83)
	8.45 (3.18)	8.15 (3.06)	8.86 (3.33)
Pros (3 -15)			
Concentration ^d^	2.01	1.80	2.31
Relaxation	3.09	3.02	3.20
Tension	3.34	3.33	3.35

Cons (3 -15)^c^	9.66 (2.89)	10.34 (2.75)	8.72 (2.83)
Embarrassed ^d^	2.18	2.48	1.79
Foolish ^d^	3.48	3.74	3.11
Bothers	3.93	4.07	3.73

*Note*. Decisional Balance scores by gender. Pros, Score on “Pros” Subscale; Cons, Score on “cons” Subscale;

a Minimum and maximum scores appear in parentheses after subscale name

b Mean (standard deviation)

c Significant differences between men and women based on results of t-test; p <0.0005

d Significant differences between men and women based on results of t-test; p <0.01 .

**Table 3:** Regression Analyses for Hypothesized Prediction: Preparation Stage.

Covariate	R^2^	Multiple R^2^	β	sr^2^	p-value
Step 1 Gender	0.2740	0.2740	3.94	0.99	<0.0002
Step 2 Pack-years	0.1253	0.3993	0.072	0.02	<0.0006
Step 3 Smoke Quit Attempt	0.05	0.45	−0.08	0.04	<0.029

*Note.* n = 55. β = standardized beta weight; Smoke Quit Attempt, Lifetime Quit Attempts.
